# Understanding uptake of continuous quality improvement in Indigenous primary health care: lessons from a multi-site case study of the Audit and Best Practice for Chronic Disease project

**DOI:** 10.1186/1748-5908-5-21

**Published:** 2010-03-13

**Authors:** Karen L Gardner, Michelle Dowden, Samantha Togni, Ross Bailie

**Affiliations:** 1 Australian Primary Health Care Research Institute, Australian National University, Canberra, Australia; 2 Menzies School of Health Research, Darwin, Australia

## Abstract

**Background:**

Experimentation with continuous quality improvement (CQI) processes is well underway in Indigenous Australian primary health care. To date, little research into how health organizations take up, support, and embed these complex innovations is available on which services can draw to inform implementation. In this paper, we examine the practices and processes in the policy and organisational contexts, and aim to explore the ways in which they interact to support and/or hinder services' participation in a large scale Indigenous primary health care CQI program.

**Methods:**

We took a theory-driven approach, drawing on literature on the theory and effectiveness of CQI systems and the Greenhalgh diffusion of innovation framework. Data included routinely collected regional and service profile data; uptake of tools and progress through the first CQI cycle, and data collected quarterly from hub coordinators on their perceptions of barriers and enablers. A total of 48 interviews were also conducted with key people involved in the development, dissemination, and implementation of the Audit and Best Practice for Chronic Disease (ABCD) project. We compiled the various data, conducted thematic analyses, and developed an in-depth narrative account of the processes of uptake and diffusion into services.

**Results:**

Uptake of CQI was a complex and messy process that happened in fits and starts, was often characterised by conflicts and tensions, and was iterative, reactive, and transformational. Despite initial enthusiasm, the mixed successes during the first cycle were associated with the interaction of features of the environment, the service, the quality improvement process, and the stakeholders, which operated to produce a set of circumstances that either inhibited or enabled the process of change. Organisations had different levels of capacity to mobilize resources that could shift the balance toward supporting implementation. Different forms of leadership and organisational linkages were critical to success. The Greenhalgh framework provided a useful starting point for investigation, but we believe it is more a descriptive than explanatory model. As such, it has limitations in the extent to which it could assist us in understanding the interactions of the practices and processes that we observed at different levels of the system.

**Summary:**

Taking up CQI involved engaging multiple stakeholders in new relationships that could support services to construct shared meaning and purpose, operationalise key concepts and tools, and develop and embed new practices into services systems and routines. Promoting quality improvement requires a system approach and organization-wide commitment. At the organization level, a formal high-level mandate, leadership at all levels, and resources to support implementation are needed. At the broader system level, governance arrangements that can fulfil a number of policy objectives related to articulating the linkages between CQI and other aspects of the regulatory, financing, and performance frameworks within the health system would help define a role and vision for quality improvement.

## Background

Experimentation with continuous quality improvement (CQI) processes is well underway in Australian primary health care, particularly in Indigenous services where there is considerable interest in using these methods to improve the delivery of a range of core primary health care services [1]. These efforts are linked at the policy level to investment in processes and mechanisms that aim to improve the standard and quality of care delivered across the spectrum of treatment, prevention, and promotion activities, and to improve access, efficiency, and safety. While a number of quality initiatives are currently being employed by services, and there is growing experience with implementation in different settings and contexts, little research into how health organizations take up, support, and embed complex innovations like CQI is available on which services can draw [2]. In the Australian setting, this may be because of the limited history with experimentation, but more broadly it is also associated with the methods that have traditionally been used to study the effectiveness of complex interventions like CQI--experimental designs that focus on measuring outcomes but are blind to the study of the innovation itself, the contexts into which they are introduced, and the processes of implementation that are utilized [3,4]. Not only are these methods inadequate for explaining variation in outcomes and enabling the transferability of results between settings [5,6], they have also resulted in a paucity of robust methodological approaches that can produce analyses useful for informing implementation in the policy and practice worlds. CQI processes are complex interventions that raise technical and administrative challenges and involve subsequent changes to roles, relationships, and routines within organizations in different locations and levels in the system. Understanding these changes, and how organizations deal with them to succeed in implementation, involves the systematic analysis of the development, uptake, and implementation of innovations within their specific contexts.

In this paper, we examine the practices and processes in the policy and organisational contexts that support and/or hinder services' participation in a large scale primary health care quality improvement program. We aim to explore the dynamic interaction of these practices with the particular features of the Indigenous primary health care service environment. Our focus is confined to the initial year of engagement, during which decisions to take up and implement the quality improvement program were first made and organisations moved to implement the system. Our main interest is in understanding the key drivers so that lessons for informing the development of more effective strategies for supporting uptake can be developed.

The program, known as the Audit and Best Practice for Chronic Disease (ABCD) project, began as a demonstration project in 12 Indigenous primary health care services in the Northern Territory in 2002 and has since spread through an extension phase to almost 70 Aboriginal health services in four states and territories. It is an action research project that investigates the impact of organisational systems on the quality of chronic disease care and outcomes for clients. Participating organisations in each jurisdiction employ their own hub coordinator who provides a support and coordination role for that jurisdiction. Formal participation agreements set out the roles and responsibilities of the parties and services undertake to participate in at least three full annual CQI cycles over the life of the extension phase. In return they are able to utilize ABCD audit tools, have their data analysed through the real-time web based system, receive implementation support and participate in a network of ABCD services. Approximately 60 additional services have used the project tools and processes without being formally enrolled in the research project, and it is likely that more services would have joined the research project had funds for hub coordinators been available in other jurisdictions. Ethics approval from research ethics committees in each jurisdiction was obtained.

Like other CQI approaches, ABCD aims to facilitate ongoing improvement by using objective information to analyse and improve systems and service delivery [7]. Participating services use annual quality improvement cycles (plan-do-study-act) and a set of clinical audit and system assessment tools to measure the quality of their systems and service delivery in relation to recognized best practice. This information is used to develop action plans that can lead to improvement. Details of the study protocol [8] and the impacts on care delivery [9] and client outcomes [10] have been published elsewhere. In this paper, we focus on factors influencing uptake and establishment of the CQI processes into services in the first cycle.

## Methods

We used a mixed method approach across sites participating in the extension phase of ABCD. Sites consist of a regional organization, either an Aboriginal community controlled health corporation and its primary health care services or a government department and the primary health care centres it operates in each region. The paper draws on routinely collected data describing regional and service profiles, uptake of tools, and initial progress through the first CQI cycle; as well as data provided quarterly by hub coordinators in each region about their perceptions of the local level barriers and facilitators to participation. These data were collected in a common structured format and complemented with semi-structured in-depth interview data, as well as data obtained through observation and document review.

### Study setting and progress through the first cycle

**Figure 1:**
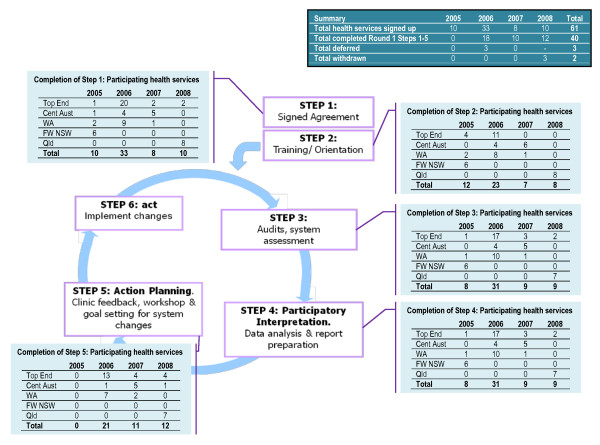
**Number of participating health services completing round 1 ABCD cycle between 1 January 2005 and 30 November 2008**.

Aboriginal health services in the Northern Territory, Western Australia, NSW and Queensland participated in the ABCD extension phase that ran from January 2006 to December 2009. We report on 61 of these services, for which data were available between the period January 2006 and December 2008. Enrolment into the project was ongoing throughout the period, with most (33) services joining during 2006, as shown in Figure 1. Thirty-five services are 'community controlled', that is they are non-government organizations usually run by Indigenous corporations that have CEOs and are governed by community boards. The remaining services are government-run, the majority of which are in the Northern Territory and Queensland. About one-third of all services are accredited (36%). Staffing profiles differ dramatically according to the service location and the size of the populations they serve (range from around 33,000 in metropolitan areas to less than 100 in remote locations). Some remote services, for example, have only a clinic nurse manager and an Aboriginal health worker with visiting medical and allied health services provided on a rostered basis.

Forty services (65%) completed all steps in the first cycle. This included completing the signed agreement, conducting the diabetes and preventive services clinical audits and the systems assessment, providing feedback, and conducting an action planning workshop. Of those that did not complete all steps, six services made an active decision not to follow the process as recommended, preferring to adapt the feedback component of the cycle. Others were either delayed (3) or withdrew (2). Only 26 services completed the steps in the cycle within the recommended three-month timeframe. A variety of reasons accounted for these differences, some internal to the service and organisational environments and local community, and others in the broader service system. The key influences associated with initial uptake and progress through the first cycle are discussed below. The extent to which the use of selected tools was sustained across the full three cycles of the project will be the subject of a later paper.

### Data collection and analysis

We took a theory-driven approach to inform data collection and analysis, drawing on literature on the theory and effectiveness of performance management and CQI systems [11,12], and using the Greenhalgh diffusion of innovation framework as the organizing framework for data collection, including the structured self-report data from hub coordinators and for the semi-structured interview schedules. The Greenhalgh framework was developed through a systematic review and is a multi-tiered model of uptake and implementation of complex innovation in health organizations. It identifies the key domains or areas in which factors influencing uptake and implementation are found. These are in the attributes of the innovation and the change agency within which it sits; the process of diffusion or dissemination; elements of the user system; and in the outer system context.

A total of forty-eight interviews were conducted at the study sites and with government officials and key people involved in the development and dissemination of the ABCD project. At the health service delivery end, interviews were held with regional program managers, health centre managers, and clinicians. In the policy sphere, key health bureaucrats who had some involvement in the early phase of ABCD were interviewed. In the ABCD project team, academics, the program manager and regional hub coordinators were interviewed.

Analysis of data proceeded in several related stages. The first stage involved the compilation of the service participation data and thematic analysis of the hub coordinator data. This produced a summary of progress across all sites and a list of key barriers and facilitators to uptake and ongoing participation. These were then aggregated to the regional/state level for comparison. Interview data were analysed individually according to the key themes identified in the Greenhalgh domains. We then drew on the relevant data sources to develop a more in-depth narrative account of the factors, both facilitators and barriers, to uptake and establishment of the CQI cycle in two sites. We further developed these by comparing between sites and then sought to identify the common core underlying drivers and impediments. We present our results as interpretive accounts in which we have aimed to synthesise and highlight the commonalities and differences between sites, rather than as directly comparable units of analysis, as this is clearly not possible given the diversity of contexts, organizational arrangements, and other factors that influence interactions.

## Results

### ABCD Attributes

In the series of interviews conducted for this research, we found broad support for the ABCD approach to CQI and considerable enthusiasm for the benefits that were perceived as arising from its use. There was a widespread perception that the system offered some distinct advantages over pre-existing quality approaches, training and technical support were available to assist services with implementation, and services could adapt the use of the processes and steps in the CQI cycle to suit their own environment and needs. The main initial concerns related to the amount of work that ABCD generated. Notwithstanding these concerns, much of the motivation for taking up ABCD revolved around perceptions of the need to improve accountability and a sense that ABCD provided a means of doing this. We noted variation in the different stakeholders' views about the types of accountability they perceived it offered, to whom, and for what.

#### Relative advantage

In Aboriginal community controlled organisations, leaders spoke of the drive to improve and be accountable to communities for Aboriginal health services, and to trial a method for investigating the effectiveness of the strategies and models of service being offered. They wanted to use the methodology to assess the quality of care processes, monitor progress, and evaluate the impacts of programs on health. Some were more enthusiastic than others about the potential of ABCD to do this, arguing that previous experience with quality improvement had been with short-term discrete processes like incident reporting or accreditation that did not provide a structured, ongoing approach that linked system development with care delivery and client outcomes. Others were more interested in combining the use of ABCD audit tools and quality processes with aspects of other quality improvement methods and cycles. One concern was that ABCD tools captured information that was beyond the capacity or role of services to address. Others raised these same issues but saw the information as an advantage because data could be aggregated at a regional level for analysis and addressed as part of broader policy and program processes. In government agencies, ABCD was seen as providing the tools for stimulating improvements in service delivery and as a framework for extracting data that could be aggregated for two related purposes: to monitor progress and measure the impact of the newly developed state based chronic disease strategies, and to feed into national performance reporting processes.

#### Hard core/soft periphery

The ABCD approach contains what has been termed in the research literature 'a hard core and a soft periphery' [13]. That is, the audit tools appear as the hard core or irreducible elements of the innovation, and the annual plan-do-study-act cycles, the 'soft periphery' or processes required for implementation. Innovations with these properties are thought to be taken up more readily than those without [13]. The ABCD hard core provides a standardized method for the collection of comparable data across services and has not been adapted at the service level. The soft periphery or steps in the cycle have been adapted by organizations in different ways to maximize fit in the local context and to build acceptability among staff. For example, one site did not provide feedback to services in the first year, but developed protocols for action instead. Others experimented with conducting feedback and action planning processes in different configurations. Some services also worked closely with the ABCD team to provide feedback on the practicalities of the protocols and operational definitions. While the focus during the first cycle was primarily on putting the ABCD processes into place, in later cycles this feedback became increasingly important to ensure standardization and alignment of the tools with other policy and program developments.

#### Technical support

Training and technical support provided by ABCD project staff was seen by stakeholders as critical for getting the project up and running in services. For those who joined ABCD early in the early part of the extension phase, the project manager and hub coordinators trained staff in different sites and assisted services directly with conducting audits, delivering the systems assessment, interpreting data, and giving feedback sessions. This provided a level of consistency to the collection and interpretation of audit data and the delivery of the cycle components. As the number of participating services has grown, the project has experienced difficulty in meeting demand for support. While this did not directly affect health centres that joined in the early part of the extension phase, it later became clear that new strategies were needed to support and train staff in those services joining later. The advantage for later joiners, however, was that they could draw on and gain support from the experience of the early enrolees.

#### Transfer of knowledge

Some stakeholders saw potential for transferring the knowledge gained from implementing ABCD to other tasks within the organization. Some community controlled organisations began using ABCD as the framework for evaluating new programs, developing output and intermediate outcome indicators and applying the systems assessment and feedback methodology to measuring improvement in other programs. Several services, government and community controlled, used ABCD tools to extract clinical data that were required for reporting on another government program. In some cases, there was a strong emphasis on the reporting processes as well as the quality improvement components; in others, the focus was more exclusively on extracting data for performance reporting, which appeared to lead to reduced interest in completing the quality cycle. Some services experienced confusion about the distinction between other major quality programs and ABCD, and where this occurred, collection and reporting of data were experienced as overly burdensome. There were a small number of coordinators who had a very clear understanding of the relationship between the major programs, and aligned internal service processes and routines to support their combined use.

### Active dissemination process

#### Role of expert opinion, champions and change agents

Opinion leaders [14-16] and champions [17-19] can have a strong influence on individual opinion relating to new innovation. The ABCD project team took an active approach to influencing the opinion of key stakeholders as a means of facilitating uptake of the project. After an active recruitment phase in the Northern Territory, subsequent uptake eventuated through informal spread, largely as a result of interest that was generated through presentation of research findings from the trial phase at forums and conferences, through initiation of contact with potential stakeholders, and through championing the process in medical networks. Many stakeholders at different levels of the system had to be engaged, and ABCD efforts in this regard seem to have had an important, though differential impact on influencing provider opinion. Influencing clinic managers and other clinicians was sometimes difficult, even in cases where their own organizations sought their participation. There was a widespread perception that remote area managers often operate with little support, are overworked and under resourced, and some coordinators felt that in the absence of formal agreements with their auspicing bodies, together with commitment of support, efforts to influence them were unlikely to be successful. Several different forms of influence appear to have been important in engaging the initial interest of the various stakeholders.

First, the role of expert opinion seems to have been influential in the initial engagement of senior managers, a number of whom commented on the significance of the research findings from the trial phase on their decision to proceed with ABCD. The fact that the project had demonstrated improvements in care and clinical outcomes for clients and was acceptable within the Australian Indigenous context was mentioned by numerous managers as important. This appears to have conferred a sense of legitimacy on the project and allowed prospective managers to assess the likely benefits and risks of being involved. Reflecting on this, one senior manager commented, 'ABCD gave health service managers tools and authority to adopt new ideas. Champions can be effective but you need to give people authority to act. ABCD reports, especially the impact on intermediate outcomes, were very compelling.'

The project manager and hub coordinators played a key role in the initial engagement of services and community health boards, particularly in two jurisdictions where their experience in working in Aboriginal health services and their links with communities, particularly remote ones, gave people confidence that ABCD was viable in those contexts. Their influence seemed to operate on the basis of their status as well as the personal and professional networks and relationships they had with Indigenous communities.

General practitioners (GPs) were seen as difficult to engage, and of all groups GPs were the least likely to attend the system assessment and feedback sessions. This was compounded everywhere by institutional employment arrangements that are perceived as mitigating against GP involvement in health centre team work. One jurisdiction had a senior medical champion who was perceived as very influential in engaging GPs. She was a strong advocate for ABCD, and her influence operated through peer-based medical networks where she helped to introduce quality improvement concepts and construct meaning about the purpose and role that ABCD could play in improving practice. There was great interest in all states in establishing cross-state linkages to draw on the influence of the medical champion, who subsequently delivered professional development sessions and spoke with individual GPs in other states and territories. Where GPs were enthusiastic about ABCD, they were more likely to play a role in reviewing data and developing strategies for improving care. Over time, a number of jurisdictions began actively developing strategies to build medical champions in their own regions and to address the institutional barriers to their engagement.

Nurses and Aboriginal health workers, on the other hand, were usually engaged in ABCD through the process of implementation after the decision to proceed had already been made. They developed their knowledge of ABCD through hands-on experience of conducting audits and participating in feedback and planning sessions, for which they were provided with training. Most were enthusiastic about the benefits of continuous improvement and the impact the information from auditing had on their perception of the quality of the care they provided, but a number did not see this as their role and resisted being involved. Some nurses and health workers also reported needing more information about ABCD. Unlike GPs, nurses are reported to be influenced by hierarchical networks [20], and this seemed to have been the case in ABCD where their participation followed from their role and involvement in the clinics. However, efforts to influence nurses may have benefited from a broader engagement strategy

### Organisational antecedents

There was great variation between sites in governance arrangements, infrastructure, staffing levels and continuity, leadership and management styles, as well as in the characteristics of the local communities they served. Previous studies have emphasized that organizations with absorptive capacity for new knowledge, good leadership, and management [21,22] are more likely to experience success in taking up innovations. Several characteristics seemed to have been important in explaining the rate of implementation of ABCD.

#### Absorptive capacity for new knowledge

The combination of formal expertise, technical infrastructure, organisational know-how, and informal networks make up what has been described as absorptive capacity [21,23]. These features were present in participating sites in different combinations and to varying degrees. The specific combination seemed to shape the capacity for implementation, the rate at which it proceeded, and the kinds of problems that arose.

Where there were key staff who had an interest, some experience and expertise in using data for performance improvement purposes, uptake of the tools and processes proceeded with relative ease, and there was greater enthusiasm for what could be achieved. These people had a good feel for how data could be used to underpin discussion about improvement and could see opportunities for acting on practice. Where they were in positions that allowed them to drive the process, they did so with relative independence, and where these skills also existed within the health centre team, the processes were embedded with relative speed into organisational routines. These services were less reliant on outside support, either in terms of direction or for technical expertise in relation to selecting samples for audit, applying definitional criteria, interpreting data, and providing feedback. Medical knowledge was also critical to synthesizing clinical information from different audits and interpreting results. Many coordinators drew on the expertise of the medical champion for this when they did not have a doctor centrally engaged in the process in their local area.

Well established administrative and information systems were also critical. These could either be paper-based or computerized, but where services were moving between systems, either combining the use of paper-based and computerized systems or moving from one form to another, difficulties were often experienced with finding information. This added significantly to the time required to conduct audits and sometimes affected the results of the audit, which at times led to disputes. There was ongoing discussion in most sites about the extent to which audit results for care delivery reflected omissions in documentation or in delivery of the care itself, and problems with IT could affect the quality of the data from one year to the next.

#### Leadership and management

Leadership and management were critical to successful uptake. Two important functions seem to have been carried out by program leaders in this respect. They played a key role in shaping an organisational vision for what could be achieved through investing in quality improvement and in articulating how ABCD would build capacity for achieving that. They also masterminded broad strategies for implementation and provided a mandate to proceed.

First, leaders in all jurisdictions demonstrated extensive knowledge of the local and national health care environments, and those who experienced success in achieving uptake saw the changes required for embedding CQI as structural and behavioural. They exercised judgment in how they went about motivating staff and operated at multiple levels to alter the local environment in ways that could enable staff to participate and put into place new structures and routines to support them. They did so incrementally, building on small successes and adapting and trialing different strategies, chipping away over time. In one site that had strong leadership and vision for ABCD, staff attended workshops, listened to presentations, and the Board was sent on a study tour to learn about quality improvement. ABCD was included as a standing item in regular senior management meetings, including one that became a forum for providing broad support for implementation. At a later time, the manager went to considerable lengths to employ GPs with a chronic disease focus and an interest in being involved in CQI.

Successful leaders engaged staff in building a shared organizational vision as well as in making sense of what ABCD would mean in relation to their own role. This involved discussion and debate which sometimes led to tensions and conflict. While staff in all sites spoke in general terms about improving practice and using data to see where the service was 'falling down', in one site senior staff had a shared understanding of the broad organisational agenda as well as clarity about the perceived benefits in relation to their own role. To the primary health care program managers, for example, ABCD became a method for reorienting service delivery away from an acute care model and toward a population health one. Clinic managers regarded ABCD as a business-planning tool, and ABCD became the blueprint for the service business plan. Doctors saw potential for reviewing practice arrangements through the collection and analysis of data that was sufficiently fine-tuned to demonstrate changes in clinical status. The CEO was focused more on measuring broad achievements, identifying areas for improvement, and finding ways of feeding back information to communities and boards, as well as conveying improvement to funders. Together, these accounts were complementary and provided a strong foundation for embedding ABCD into organizational routines and practices.

Where leaders did not play a central role in engaging staff in building an organizational vision or provide a high-level formal mandate to proceed, it was largely left to individual project managers to work with clinics on putting ABCD into place. This left the process more to chance and depended on the power and inclination of middle managers to support it. In one jurisdiction where an organization-wide high-level mandate appeared not to have been provided, there was limited clarity in relation to roles and function. An implementation plan was never agreed, resourcing of the project coordinators was shifted from one department to another and subsequently fell between the two, reporting structures were never formalised, and, despite enthusiasm in many places, the process hung on individual interest and goodwill.

### Organisational readiness

At the local level, readiness is thought to be influenced by tension for change [24], the relative balance of opponents and supporters [25], compatibility with existing ways of working, and project management skills [24,26]. In this study, we found a somewhat contradictory set of influences on the readiness of services to be involved in ABCD.

#### Tension for change

It is difficult to argue that tension for change does not exist in all sectors in relation to improving Indigenous health, government- and community-controlled alike. On the one hand, there is a sense of urgency that something must be done, and this has recently been fuelled by the series of reports and events surrounding the Northern Territory Intervention, the subsequent Rudd Apology, and the Closing the Gap response. Many people believed that ABCD processes could provide a stimulus for motivating staff in delivering best practice care, thereby improving the chances of maximising the benefits that services can contribute to health. Organisations are very keen to examine and demonstrate the impact of the programs they provide. On the other hand, while there is enthusiasm for this, there is also a sense of burden among staff in remote communities that sometimes serves to create a sense of hopelessness and leads to inertia. The processes that produce this have been described in detail elsewhere [27]. The poverty in which Indigenous people live in these communities, the constant flow of staff in and out, the lack of apparent improvement year in and year out, the constant on-call, the long working hours, and the uncertainty surrounding the best way to intervene was described by people in this study. It is a significant problem that grinds people down and focuses them on meeting their obligations for core tasks only. Ironically, in places where one might expect the tension for change to be greatest, the capacity for introducing it may be lowest: 'I think ABCD is a great idea. If I could get time to do it, it might even make my staff stay.' However, we also found examples where centre managers were well supported and, despite the extraordinary demands experienced in their daily work, had leadership and management skills that worked to motivate change and get programs implemented.

#### Compatibility with existing health centre systems and processes

Health centre managers reported excellent fit between the ABCD tools and pre-existing service delivery and administrative systems for chronic disease care delivery, particularly in relation to recall, care planning, and recordkeeping. There was prior experience of using disease management guidelines, and care planning had already introduced notions of inter-disciplinary teamwork, review, and goal setting. Audit tools could equally be applied in paper-based or computerized systems and, as most services are moving toward computerization, this was seen to be essential. Because there is no single integrated data reporting system in most community health settings, implementing a standardized, automated quality system that extracts data from a comprehensive set of records is not yet possible. ABCD audits are conducted on files of 30 randomly selected patients each year, and in most health centres this involves examination of paper-based as well as computerized records in several different systems for a single client. Although cumbersome, this method gives a good overall indication of the level of service delivery, and in services that have adequate staffing levels this system is seen as acceptable. For others, particularly in remote areas where staff turnover is high, core positions are often vacant or filled by agency staff, the audit system is seen as unrealistic and a major barrier to ongoing implementation by many managers. These services are particularly reliant on external support to coordinate and implement auditing, feedback, and action-planning processes.

#### Power balances-supporters verses opponents

While many people embraced ABCD with enthusiasm and interest, there was also ambivalence in some places. This seemed mostly to be associated with competing pressures and demands, rather than with any direct opposition to the ABCD concept itself. There were the usual debates and concerns that could be anticipated in any uptake process. For example, many people expressed concern in the early stages that auditing was about policing services and checking up, but these were rapidly dispelled and did not persist. However, when opposition did occur it was usually manifest in refusal to participate in the process, in disputes over the validity of the sample drawn for audit, or in the validity of the data itself. Opposition from different sources tended to either block or delay progress at different points. For example, commitment from the clinic manager was critical to putting the processes into place. Where responsibility for overseeing and implementing the cycle fell to the manager, and there was no coordinator or consistent staff to assist, clinic managers sometimes did not want to take up ABCD, and the project did not go ahead. Even withstanding the efforts of the hub coordinators to train staff, assist services to conduct audits, run feedback sessions, and help with action plans, where there was ambivalence on the part of the centre manager and it was not made a priority, implementation of the cycle tended to stagnate or be delayed. In other cases, services signed up to participate and were overtaken by problems in the community or with staffing and withdrew in the next cycle. Many government staff in one jurisdiction believed that implementing ABCD into remote clinics was not viable without the commitment of additional resources. Where opposition came from clinical staff, implementation of actions that could lead to improvements in care was more likely to be affected. Action plans were generally embedded into services through team processes, such as by addressing matters at weekly team meetings. Where there was opposition, it was less likely that follow-up of clinical or administrative issues would occur. It was clear that clinic teams needed to embed ABCD action plans into service routines, and that this needed to be supported and driven by someone in the clinic. Where there was support from a manager or senior clinician or more supporters than opponents, this proceeded more rapidly.

#### Project management

All sites had project management skills available, and those responsible for implementing ABCD at the clinic level were usually chronic disease or quality coordinators who generally had a cluster of around four to six clinics for which they developed implementation plans, coordinated staff to conduct audits, organized systems assessment meetings, wrote reports, and assisted managers with developing action plans. In some sites, they were hampered by opposition or ambivalence from clinic staff, persistent staff turnover, or lack of resources for backfilling clinics when attendance at feedback and action planning sessions was needed. This caused delays and interrupted progress through the cycle. Where there was little support and no formal response from regional or central management to clinic reports, and the drive to implement the process was left to individuals, enthusiasm for implementation sometimes dissipated and people began to argue that the organization wasn't committed, or that implementation was not viable under current service conditions.

### Initial establishment into clinics

A number of influences supported the initial establishment of ABCD into services. Those most important for establishing the first cycle related to the organizational approach to change, dedication of resources, hands-on support, and the extent of devolution of decision making.

#### Approach to change

In a number of organisations, vision was accompanied by a mandate to proceed and a clear framework for implementation that could support good project management. This included the identification of a budget stream, lines of management responsibility and reporting and a structure to which the data would come for review and response. In these sites, leaders set up internal structures, usually committees, to support implementation and where they worked well, they brought people from different places in the system together to discuss progress, examine results, construct interpretations, and debate what needed to happen next. This served several important functions. It created the sense of a shared purpose, reduced the sense of isolation that seemed to be common among clinic managers, and linked the levels of the system so that information flows were maximized. It also increased debate, which promoted understanding and meant that ideas could be shared and problems dealt with in an incremental and adaptive way and within a broader sphere of influence than was otherwise possible. These organisations displayed a sense that things could be done. Where this kind of approach was not adopted, the way of working was more task-oriented and narrowly focused. While project managers did their best to implement processes into clinics--and some achieved a great deal of success in stimulating enthusiasm and getting the cycle done during the establishment phase, where no supportive structures were established to facilitate linkages within organisations--it was difficult to achieve the same influence over the multiplicity of factors that had to be addressed.

#### Dedication of resources

All organisations invested resources into implementation by supporting project management/coordination roles, and in many sites resources were made available for backfilling staff positions that enabled them to participate in auditing, feedback, and action-planning sessions. Bigger clinics tended to have more human and other resources that could be cobbled together at times when needed to assist with implementation of the steps in the cycle. Among smaller clinics in remote locations that are hundreds of kilometers from towns, there are fewer opportunities for this, great demands in terms of the general day-to-day operations relating to service delivery, maintenance and infrastructure, and high staff turnover. In some instances, staff turnover was reported to have been complete between the time of audit and the action planning session. A number of coordinators were frustrated by this and felt that it was essential to complete the cycle in the three-month period to build on the impetus that was inevitably created when staff participated in auditing medical records. In these centres, clinic managers felt the ABCD system was unrealistic unless entirely supported by an outside team. Some of these clinic managers summed up their experiences: 'The ABCD principle is good. The workload is too high. It isn't feasible.'

#### Hands-on approach

Hands-on approach worked well everywhere. Most coordinators encouraged health centre staff to do at least some audits, and these had a dramatic impact on people's understanding of what best practice was and what quality improvement was aiming to achieve at the clinical level. Most importantly, it gave people a point of reference for thinking about their own practice. Everyone spoke enthusiastically about the benefits of this educative process. One manager commented, 'It has improved recording...We are much better on paper. And it has raised awareness about what is best practice. It's a point of reference and there isn't anywhere else to pick that up.'

#### Decision making

The literature provides some evidence that decision making needs to be devolved to the service level to facilitate uptake [28,29], and in relation to CQI, local control over interpretation of data and the development of actions to address these are seen as critical for stimulating improvement [11]. In some sites, hub coordinators retained responsibility for selecting population samples, coordinating the conduct of audits and the feedback, and action-planning sessions in the clinics with which they worked. During the establishment phase, their main focus was on introducing the key concepts and putting the steps of the cycle into action. This was not driven at the health centre level, and it was only after experience through several cycles and in some cases that centre managers, nurses, doctors, and health workers got involved in interpreting data and developing and driving action plans. This seems to have occurred more readily in services that had stable staff teams, support from the clinic manager, good clinical relationships, links with the local community, and staff with knowledge and experience in using quality improvement processes. In some places, control remained entirely centralized, staff perceived that ABCD was a regional concern, and they did not engage in any meaningful discussions about the way they went about their work.

### Outer system context

At the system level, beyond the immediate service context, the broader policy and program developments at the national and state levels provided a conducive backdrop for developing and taking up ABCD. For a number of reasons, the time was right for ABCD. First, in the policy arena, the mandate to focus attention on chronic disease had been growing since the 1990s when chronic diseases were first made national priorities and a range of policy measures and nationally funded payment arrangements began operating to promote and support best practice care delivery for chronic disease. These included the endorsement of national clinical guidelines for diabetes care, the release of the national chronic disease policy, the National Strategic Framework in Aboriginal and Torres Strait Islander Health, and a number of state-based chronic disease strategies. In addition, a series of special practice incentive payments delivered under Medicare to promote adherence to national clinical guidelines, cycles of care, and involvement of practice nurses were introduced. These have more recently been followed by additional Medicare items that support the delivery of preventive health checks for both Indigenous and non- Indigenous people.

There has also been an increasing policy emphasis on the use of performance information to drive improvements in the quality and outcomes of care [30]. The Aboriginal and Torres Strait Islander Health Performance Framework provided the framework for integrating performance reporting processes and linking these to policy processes [31]. At the service level, experimentation with quality improvement projects and accreditation is a relatively new development, followed most recently by the introduction in 2005 of a major quality initiative, the Healthy for Life program [1]. Healthy for Life is central to government efforts to improve and monitor progress toward best practice delivery of Indigenous primary health. These latter initiatives require services to collect and report a range of performance data on intermediate client outcomes and processes of care. ABCD tools and processes provide a comprehensive framework and method for collecting this kind of performance data, and a number of participating organizations in different states and territories utilize the tools for this purpose. Uptake of the ABCD system has been shown to result in increased compliance with guidelines for disease management and more consistent use of care plans [32,9]. This can potentially increase the number of clients with completed cycles of care and for eligible services, lead to increased Medicare incentive payments, which are currently low [33] and service income. In this way, ABCD has helped to build service capacity for addressing policy developments in primary health care.

## Discussion

While the literature is punctuated with inconsistencies in the use of terms like 'adoption', 'uptake', 'spread', 'diffusion', and 'dissemination' [2,34], our primary concern was with the practices and processes through which self-selecting organisations were motivated to take up and able to support the establishment into services of ABCD tools and processes. In the context of large organisations that manage a number of health services within a region or regions, this was not a discrete decision or event but a complex process that involved engaging multiple players within a web of relationships and processes that had to be negotiated and defined. We found that the process was messy and non-linear, it happened in fits and starts over an extended period-sometimes more than a year. The process was often characterised by conflicts and tensions. It had more in common with the messy model of assimilation described by Van de Ven [29] in which organisations 'moved back and forth between initiation, development, and implementation variously punctuated by shocks, setbacks, and surprises' than with the earlier stage based approaches that emphasised knowledge awareness, evaluation-choice, and adoption-implementation, such as described by Meyer and Goe [19]. Much of what we witnessed pointed to a process of change that was iterative, and reactive involving interactions between features of the environment, the service, the quality improvement process, and the stakeholders. Our findings suggest that despite initial and widespread enthusiasm for the ABCD model of quality improvement, the mixed successes of uptake and diffusion into services during the first cycle were associated with the ways in which these factors interacted in particular organisations to produce a set of circumstances that either inhibited or enabled the process of change. Organisations had different levels of capacity to mobilize resources that could shift the balance toward supporting implementation.

Many features of the Indigenous primary health care service environment would seem to mitigate against the successful uptake of innovations like ABCD. High among these was the turnover and shortage of staff in many Indigenous primary health care services, and in remote areas the additional problems of geographic isolation, poverty, and burden of illness and disease within communities is an added dimension that is unparalleled in other parts of Australia. In the service context these problems have multiple effects, not only on demand for services but also on staff morale, recruitment, retention, and workforce arrangements, many of which are beyond the capacity of individual services to directly address. While staff turnover did not appear to impede motivation for uptake, it constrained, and at times disrupted, the speed and depth with which incorporation into services could proceed. This pointed to a need for organisations to respond to quality improvement as complex system issues that have to be addressed at multiple levels of the service system.

**Figure 2:**
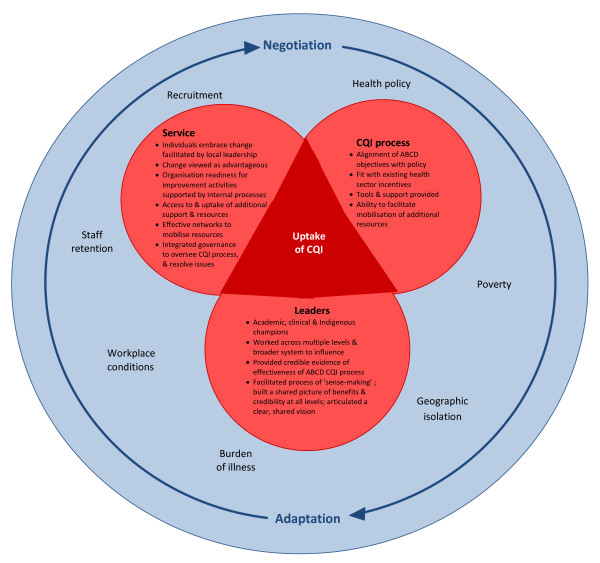
**Health Service environment: Characteristics that enabled uptake of ABCD CQI process**.

In most cases, the fact that the many difficulties did not disrupt the establishment of ABCD quality improvement processes during the first year was testament to the motivation of individuals to embrace change that they perceived as advantageous, and the readiness of organisations for improvement activities. It was also related to the alignment of ABCD program objectives for quality improvement with those in the service sector and broader policy environment, and the compatibility and fit of the tools and processes with existing incentive and regulatory frameworks and service systems. The necessary skills, information infrastructure, and resources that were needed to support ABCD were available in the sector but were differentially distributed between organisations, and there was finite capacity in the project team to provide support, training, and facilitation to assist with implementation of the cycle. Over time, as the tools were put to use, the service landscape changed and the relationships that supported this fit began to shift, new difficulties emerged that had to be addressed through an ongoing process of negotiation and adaptation (see Figure 2).

At the organisational level, capacity to influence and mediate the impact of these many factors on uptake appeared to be related to the adopted approach to change, the quality of leadership, and the extent of network connections. In this early period, the primary organisational tasks had much in common with any change management process which, as Leatherman [35] reminds us with respect to CQI, is dependent on establishing 'clarity of intent, shared goals, explicit definition of resource requirements, and stability of purpose.' Organisational leaders, usually senior managers, played a critical role in achieving these conditions, and in general they worked across multiple levels of their organization and in the broader system to influence opinion and promote uptake. There were other types of leaders also: those who championed the project amongst their professional peers and those who brought credible evidence of the effectiveness of the project. There was an academic champion, a clinical GP champion, and Indigenous champions, each of whom was involved in the project management as well as in different capacities within the project or one of its participating organisations. While leaders are known to play distinct roles, and their influence operates in different ways and through different channels [16], they all had a vision for ABCD, and they engaged in dialogue with others to promote it. This enhanced capacity for understanding, and contributed to building a shared picture of how ABCD would contribute to organizational, professional, or community objectives. It helped people to clarify their role and function. This process has been described by Weik [36] as the process of sense-making, a phenomenon that involves a capacity for 'structuring the unknown' and interacting with others in the pursuit of developing a shared meaning. According to agency theory this has distinct dimensions: imagining a future through re-evaluating the past and taking action in the present [37]. This implies that successful leadership is at least based on extensive knowledge and experience, and involves creativity and situational judgement. Leaders told stories about how ABCD could work and, based on their experience, knowledge, and status, they brought credibility and conferred legitimacy to the processes. This gave people confidence and authority to proceed. This kind of leadership was evident in pockets in different parts of the system, and the extent to which organisations could benefit from this was related to the external linkages they had with others. In this respect, the ABCD team played an important role in connecting stakeholders so that these kinds of resources could be mobilized. Leadership in itself however, was insufficient for achieving uptake. New practices and routines also had to be taken up and embedded into service systems. The extent to which organisations could do this was, in part, dependent on the internal processes they set up to support it.

Establishing quality improvement processes required services to engage in new forms of dialogue and interaction. Internal linkages were required to support this. Where organisations established or expanded high-level committee structures to incorporate oversight of ABCD and involved staff from different levels of the system, including clinical managers and those with coordination roles or responsibilities for implementation, there was greater opportunity for dialogue and broader scope for creating meaning, identifying problems and opportunities, and addressing them as they arose. Information flows were increased, service routines could be identified for adaptation, and data from services could come for review and debate. This invested it with meaning and increased the possibility that services would get a response to their data, and that organization-wide strategies could be adopted to support and address features of the broader service environment that impacted on services. It also increased accountability. In some cases, no formal linkages were created to support ABCD information flows between the central agency and the services, and this impeded the development of a dynamic and interactive process that could address the many challenges.

### Greenhalgh framework

We found that the Greenhalgh diffusion of innovation framework provided a useful starting point for investigating the many practices and processes that operate at multiple levels of the health system to impede and enhance change processes. It neatly maps the complex terrain across which the kinds of attributes, activities, processes, and practices that have been associated with uptake of innovation can be found. However, the framework is an 'aide memoir' and not a theory of implementation, which we found was essential for explaining the complex interactions of the tools and processes with the individual and team practices, organisational structures, and broader system context that we observed. In this regard, the framework is more descriptive than explanatory, with a focus on the components of a change process rather than mechanisms that might explain change or lack of change in different settings. This had several important limitations for our research.

First, we suggest that embedded in the framework are assumptions of a sequential process of change that is seen to operate from the conception of an idea through to its development, diffusion, adoption, and implementation. Our findings did not always confirm this kind of process. Change was often reactive, contested, and not always predictable. Embarking on a process of adopting ABCD revealed new ways of seeing strengths, weaknesses, and possibilities, and the starting points for engineering change differed between settings. It took people time to develop understanding and make meaning out of what was needed to create change, and this happened in an iterative way. Services were responsive and could therefore be engaged simultaneously in different aspects of addressing what is defined in the framework as attributes of 'system readiness' and 'implementation', and efforts could be derailed suddenly by changes in leadership, staff, problems in the community, or other internal or external events. The presence of a regional approach, a transformational leadership style, a problem-solving approach, good relationships, internal avenues for communication, and external linkages seemed to increase the possibility that organisations could respond to challenges and succeed in keeping an approach to uptake in place. Change was therefore more dynamic and less ordered than the framework implies. Elucidating the nature of these interactions and the more fundamental characteristics of human practice that allowed organisations in different states of 'readiness' to cope with the conflicts and transitions that inevitably occurred will be important for further understanding the critical mechanisms at play.

Second, the outer context section of the framework is underdeveloped and not sufficiently defined to take account of the nature and complexity of the dynamic interactions between these elements and their interaction with those in the 'user system'. We observed that the 'socio-political context' shaped the responses of different organisations to implementation in different ways, and had differential impacts at different points in the process. Instability in the workforce, for example, did not appear to affect motivation and interest in taking up ABCD, but it certainly inhibited internal service capacity to complete the cycles at a later time. Similarly, the tasks associated with taking up a CQI process may be similar for government and community controlled organisations alike, but the processes required to support and legitimate them internally are different and may set an organisation on a different pathway to implementation. We also observed that different professional groups have different levels of power to shape the uptake process, and attempts to engage them are arguably fashioned in ways that take account of that power and subsequently privilege some negotiations over others. Further development of the framework to incorporate this dimension may lead to a better understanding of the different drivers for uptake, and help organisations seeking to spread innovations determine the best ways to adapt their processes to achieve better engagement and uptake.

## Summary

Uptake of CQI is a complex process that involves engaging multiple stakeholders in new relationships that can support services to construct shared meaning and purpose, operationalise key concepts and tools, and develop and embed new practices into service systems and routines. Some clear messages for health authorities interested in implementing quality improvement systems emerge from this study. First, promoting quality improvement requires a system approach and organization-wide commitment. At the organization level, a formal high-level mandate, leadership at all levels, and resources to support implementation are needed. Leadership is critical to success and strategies for training and mentoring leaders are needed. Opportunities for engaging and developing clinical, Indigenous, and academic leaders and champions should be a priority to help communities and services develop a vision for quality improvement. Regional level facilitators are also needed to support services to implement the quality cycle and at the clinic level, leadership is essential to ensure that new practices and ways of working are embedded into service routines. At the broader system level, governance arrangements that can fulfil a number of policy objectives in relation to articulating the linkages between CQI and other aspects of the regulatory, financing, and performance frameworks within the health system would help define a role and vision for quality improvement. This would need to determine the parameters for data use, ownership, control, and reporting to third parties. Ongoing alignment of policies and incentives related to quality improvement and performance reporting will be critical.

## Competing interests

The authors declare that they have no competing interests.

## Authors' contributions

KG developed the original idea for the paper, conducted the interviews, analysed interview data and data on uptake and cycle completion, and wrote the early draft. MD and RB contributed ideas, assisted with several key interviews, and provided comments on the draft. ST provided comments on the draft and prepared the diagrams. All authors read and approved the final manuscript.

## References

[B1] Bailie R Sibthorpe B Gardner K Si D Quality improvement in Indigenous primary health care: History, current initiatives and future directions Australian Journal of Primary Health 2008 14 2 53 57 10.1071/PY08022

[B2] Greenhalgh TRG MacFarlane F Bate P Kyriakidou O Diffusion of Innovations in Service Organisations: Systematic Review and Recommendations The Milbank Quarterly 2004 82 4 581 629 10.1111/j.0887-378X.2004.00325.x 15595944 PMC2690184

[B3] Øvretveit JGD Evaluation of quality improvement programmes Qual Saf Health Care 2002 11 270 275 10.1136/qhc.11.3.270 12486994 PMC1743631

[B4] Walshe K Understanding what works -and why- in quality improvement:the need for theory-driven evaluation International Journal for Quality in Health Care 2007 19 2 57 59 10.1093/intqhc/mzm004 17337518

[B5] Grol R Hulscher M Eccles M Wensing M Planning and Studying Improvement in Patient Care: The Use of Theoretical Perspectives Milbank Quarterly 2007 85 1 93 138 10.1111/j.1468-0009.2007.00478.x 17319808 PMC2690312

[B6] Shortell S Bennett CL Byck GR Assessing the Impact of Continuous Quality Improvement on Clinical Practice: What It Will Take to Accelerate Progress The Milbank Quarterly 1998 76 4 593 624 10.1111/1468-0009.00107 9879304 PMC2751103

[B7] Graham C Quality in healthcare: theory, application and evolution 1995 Gaithersburg, Aspen

[B8] Bailie R Si D Connors C Weeramanthri T Clark L Dowden M O'Donohue L Condon J Thompson S Clelland N Nagel T Gardner K Brown A Study protocol: Audit and Best Practice for Chronic Disease Extension (ABCDE) Project BMC Health Services Research 2008 8 184 10.1186/1472-6963-8-184 18799011 PMC2556328

[B9] Bailie R Si D Dowden M O'Donoghue L Connors C Robinson G Cunningham J Weeramanthri T Improving organisational systems for diabetes care in Australian Indigenous communities BMC Health Services Research 2007 7 67 10.1186/1472-6963-7-67 17480239 PMC1876220

[B10] Si DBR Dowden M O'Donoghue L Connors C Robinson G Cunningham J Condon J Weeramanthri T Delivery of preventive health services to Indigenous adults: response to a systems-oriented primary care quality improvement intervention Medical Journal of Australia 2007 187 8 453 457 17937642 10.5694/j.1326-5377.2007.tb01356.x

[B11] Freeman T Using performance indicators to improve health care quality in the public sector: a review of the literature Health Services Management Research 2002 15 126 137 10.1258/0951484021912897 12028801

[B12] Foy R Jamtvedt G Young J Grimshaw J Baker R What do we know about how to do audit and feedback? Pitfalls in applying evidence from a systematic review BMC Health Services Research 2005 5 50 10.1186/1472-6963-5-50 16011811 PMC1183206

[B13] Denis JL Hebert Y Langley A Lozeau D Trottier LH Explaining diffusion patterns for complex health care innovations Health Care Management Review 2002 27 60 73 12146784 10.1097/00004010-200207000-00007

[B14] O'Brien J Oxman A Haynes R Davis D Freemantle N Harvey E Local opinion leaders: effects on professional practice and health care outcomes Cochrane Database Systematic Reviews 2000 2 CD000125 10.1002/14651858.CD000125 10796491

[B15] Grimshaw J Eccles M Greener J Maclennan G Ibbotson T Kahan JF Is the involvement of opinion leaders in the implementation of research findings a feasible strategy? Implementation Science 2006 1 3 10.1186/1748-5908-1-3 16722572 PMC1436013

[B16] Locock L Dopson S Chambers D Gabbay J Understanding the role of opinion leaders in improving clinical effectiveness Social Science & Medicine 2001 53 745 757 10.1016/s0277-9536(00)00387-7 11511050

[B17] Backer TE Rogers EM Diffusion of innovations theory and worksite AIDS programs Journal of Health Communication 1998 3 17 28 10.1080/108107398127481 10947372

[B18] Markham SK A longitudinal examination of how champions influence others to support their projects Journal of Product Innovation Management Learning 1998 15 490 504 10.1016/S0737-6782(98)00031-9

[B19] Meyer AD Goes JB Organisational assimiliation of innovations: a multi-level contextual analysis Academy of Management Review 1988 31 897 923 10.2307/256344

[B20] West E Barron DN Dowsett J Newton JN Hierarchies and cliques in the social networks of health care professionals: implications for the design of dissemination strategies Social Science & Medicine 1999 48 633 646 10.1016/s0277-9536(98)00361-x 10080364

[B21] Barnsley J Lemieux-Charles L McKinney MM Integrating learning into integrated delivery systems Health Care Management Review 1998 23 18 28 9494817 10.1097/00004010-199801000-00003

[B22] Zahra AS George G Absorptive capacity: a review, reconceptualization and extension Academy of Management Review 2002 27 185 203 10.2307/4134351

[B23] Ferlie E Gabbay J Fitzgerald L Locock L Dopson S Evidence-based medicine and organisational change: an overview of some recent qualitative research Ashburner Le:Organisational Behaviour and Organisational Studies in Health Care: Reflections on the future 2001 Basingstoke Palgrove

[B24] Gustafson DH Sainfort F Eichler M Adams L Bisognano M Steudel H Developing and testing a model to predict outcomes of organizational change Health Services Research 2003 38 751 776 10.1111/1475-6773.00143 12785571 PMC1360903

[B25] Champagne F Denis J Pineault R Contandriopoulos A Structural and political models of analysis of the introduction of an innovation in organizations: the case of the change in the method of payment of physicians in long-term care hospitals Health Services Management Research 1991 4 94 111 10115542 10.1177/095148489100400203

[B26] Rogers EM Diffusion of innovations 1995 New York: Free Press

[B27] Lea T Bureaucrats and Bleeding Hearts: Aboriginal Health in Northern Australia 2008 UNSW Press

[B28] Meyers PW Sivakumar K Nakata C Implementation of industrial process innovations: factors, effects, and marketing implications Journal of Product Innovation Management 1999 16 295 311 10.1016/S0737-6782(98)00044-7

[B29] Ven AH Van de Polley DE Garud R Venkataraman S The Innovation Journey 1999 Oxford: Oxford University Press

[B30] Smylie J Anderson I Ratima S Crengle M Indigenous health performance measurement systems in Canada, Australia, and New Zealand The Lancet 2007 9527 367 2029 2031 10.1016/S0140-6736(06)68893-4 16782494

[B31] Anderson I Recent developments in national Aboriginal and Torres Strait Islander health strategy Australia and New Zealand Health Policy 2004 1 3 10.1186/1743-8462-1-3 15679932 PMC544962

[B32] Si D Bailie R Connors C Dowden M Stewart A Robinson G Cunningham J Weeramanthri T Assessing health centre systems for guiding improvement in diabetes care BMC Health Services Research 2005 5 56 10.1186/1472-6963-5-56 16117836 PMC1208882

[B33] Wilkinson D Mott K Morey S Beilby J Price K Best J McElroy H Pluck S Eley V Evaluation of the Enhanced Primary Care (EPC) Medicare Benefits Schedule (MBS) Items and the General Practice Education, Support and Community Linkages Program (GPESCL) -- final report Canberra Australian Government Department of Health and Ageing

[B34] Rye C Kimberly JR The adoption of innovations by provider organizations in health care Medical Care Research and Review 2007 64 3 235 278 10.1177/1077558707299865 17507458

[B35] Leatherman S Optimizing quality collaboratives Qual Saf Health Care 2002 11 307 10.1136/qhc.11.4.307 12468688 PMC1758014

[B36] Weik K Sensemaking in Organisations 1995 Sage

[B37] Emirbayer M Mische A What is agency? The American Journal of Sociology 1998 103 4 962 1023 10.1086/231294

